# Video-delivered emotion-focused mindfulness therapy for late- life anxiety: study protocol for a feasibility randomized controlled trial

**DOI:** 10.1186/s40814-021-00905-0

**Published:** 2021-09-03

**Authors:** Stacey Hatch, Dorothy Kessler, Marcia Finlayson, Soham Rej

**Affiliations:** 1 Aging and Health Program, School of Rehabilitation Therapy, Faculty of Health Sciences, Queen’s University, 31 George St, Kingston, Ontario K7L 3N6 Canada; 2 Department of Psychiatry, Lady Davis Institute /Jewish General Hospital, McGill University, 3755 Chemin de la Côte-Sainte-Catherine, Montréal, QC H3T 1E2 Canada

**Keywords:** Late-life anxiety, Mindfulness, Meditation

## Abstract

**Background:**

The worldwide prevalence of anxiety in older adults is estimated at between 6 and 10%. In Canada, adults 65 and older experience anxiety at a rate of 6.4%, affecting more than 300,000 people. Anxiety in older adults has been linked to difficulties retaining new information and engaging in instrumental daily activities of living. Due to COVID-19 restrictions, novel methods of delivering therapy programs remotely are needed; however, data is limited. There is some evidence that older adults prefer non-pharmacological approaches for anxiety that can be delivered in the community. Emotion-focused mindfulness therapy (EFMT) is a mindfulness-based intervention that emphasizes meditation and observing thoughts and emotions as they arise. This emphasis has been demonstrated to reduce symptoms of anxiety in general populations. This study aims to evaluate the feasibility of EFMT with older adults.

**Methods:**

This pilot feasibility randomized controlled trial will use a wait list control trial design. Due to COVID-19 restrictions, we will use telehealth delivery via Zoom rather than in-person delivery. The first 48 people to meet the eligibility criteria will be randomly allocated to either receive EFMT immediately, or in approximately 8 weeks time from enrollment (1:1 allocation ratio). Data will be collected at baseline, 8 weeks following baseline, and 16 weeks following baseline. The primary outcomes will determine the feasibility of the intervention based on recruitment, enrolment, retention, and adherence to all components of the intervention. The secondary outcome will be changes to anxiety over time.

**Discussion:**

The results of this trial will determine the feasibility and potential effectiveness of video-delivered EFMT for late-life anxiety compared to no treatment. If the results are promising, a larger randomized controlled trial may be conducted.

**Trial registration:**

ClinicalTrials.gov, NCT04415528. Registered on June 4, 2020

**Protocol version:**

Protocol version 2, January 2, 2021

**Supplementary Information:**

The online version contains supplementary material available at 10.1186/s40814-021-00905-0.

## Introduction

### Background and rationale

The worldwide prevalence of anxiety in older adults is estimated at between 6 and 10% [1, 2]. However, actual numbers may be higher because anxiety frequently goes undiagnosed in adults 65 and older, although symptoms such as insomnia, irritability and agitation are reported to primary care providers [3]. This high prevalence of anxiety is of concern because anxiety is linked to decline in memory and is a leading cause of disability in older adults [4–7]. In a study of 79 older adults, those with anxiety showed a greater decline in memory over a 4-year period [5]. The worldwide prevalence of cognitive impairments has been estimated at between 10 and 20% in people over 65 [8–13]. Mild cognitive impairment (MCI) can be understandably troubling for those living with the condition because MCI may be a prodromal symptom of dementia, with up to 15% of people progressing to dementia [14].

Anxiety in older adults has also been linked to negative effects on quality of life, problems managing stress, difficulty retaining new information, and sleep disturbances [15–17]. Older adults who self-report anxiety, depression, or both also experience a decline in functional activities, difficulties with the instrumental activities of daily living, increased mortality due to illness, suicidal ideation particularly among men, and increased use of health care services [3, 18]. Present treatments for anxiety in adults 55 and older have met with limited success. While pharmacotherapy is frequently used to manage symptoms of anxiety in older adults, this approach has been related to negative side effects such as cardiovascular issues and falls [3, 19–22]. There is evidence in the research literature to suggest that older adults favor non-pharmacotherapy approaches, such as psychotherapy, but do not always have access to public mental health care, or can afford private mental health care [3, 23]. Given the increased rates of anxiety due to COVID-19, there is an identified urgent need for evidence based alternatives to pharmacotherapy for the treatment of anxiety that are feasible, potentially scalable, and can be delivered at the primary care and community levels [23, 24].

Mindfulness-based interventions (MBIs) in the treatment of anxiety, depression, or anxiety comorbid with depression are an emerging area of research interest [25–29]. MBIs offer a guided non-judgemental approach to attending to one’s lived experiences through techniques such as meditation and psychoeducation [30–32]. Other techniques include cultivating a focus on the present moment with self-compassion [30–32]. Emotion-focused mindfulness therapy (EFMT) is a MBI that emphasizes meditation, and observing thoughts and emotions as they arise [28]. EFMT has been demonstrated to reduce symptoms of anxiety in general populations [33, 34]. EFMT’s focus on meditation and the felt sense of emotions, rather than learning new material, makes it a promising intervention for reducing symptoms of anxiety for older adults who often report normal aging problems such as general forgetfulness and difficulty with word recall [8]. EFMT has not yet been tested in older adults or community dwelling adults with anxiety. It is hypothesized that EFMT will be feasible for community dwelling older adults who experience anxiety.

## Methods

### Participants, interventions, and outcomes

This study aims to evaluate the feasibility of EFMT for community dwelling adults 55 and older with symptoms of anxiety. The secondary outcomes will be measurements of changes to anxiety, sleep, and subjective memory functioning over time.

### Objectives

Primary objectives are as follows:

1. Determine if participants can be recruited, enrolled, and retained
2. Determine if participants can adhere to all components of the intervention

Secondary objectives are as follows:

1. Examine the extent to which participants who receive the intervention have decreased levels of anxiety compared to those who do not receive the intervention
2. Examine the extent to which participants have improved sleep quality compared to those who do not receive the intervention
3. Examine the extent to which participants who receive the intervention have improved subjective memory functioning compared to those who do not receive the intervention

### Design and setting

The study is a feasibility randomized controlled trial (RCT) using a wait list control trial design. Due to COVID-19 restrictions, this study has been adapted from in-person delivery to telehealth delivery via Zoom. The first 48 people to meet the eligibility criteria will be randomly allocated to receive EFMT immediately, or in 8 weeks from time of baseline assessment (1:1 allocation ratio). Data will be collected by a blinded to group research assistant (RA) at baseline (T1), 8 weeks following baseline (T2), and 16 weeks following baseline (T3). Participants will be recruited from primary care clinics and community organizations that provide support to older adults. The principal investigator (PI)/facilitator of the EFMT groups will be blinded to the results of outcome assessments until the end of the intervention phase of the study. The PI is a registered psychotherapist certified in delivering EFMT and will facilitate all groups.

### Eligibility criteria

The PI will screen potential participants for the following inclusion criteria: participants will be community dwelling older adults 55 years of age and older with no existing therapeutic alliance with the PI; will self-report or have a diagnosis of anxiety; can speak English; and can commit to the 8-week program and three assessment phone calls with the RA. The presence of symptoms of anxiety will be assessed using the Geriatric Anxiety Inventory (GAI) with a cut off score of 10 which indicates the presence of anxiety [35]. Exclusion criteria will be having begun or stopped psychotropic medications within the previous six weeks [36]. Further exclusion criteria will be a Telephone Montreal Cognitive Assessment (T-MoCA) with a cut off score of 19 which indicates the possibility of mild cognitive impairment and would be beyond the scope of this study [37, 38]. The T-MoCA is conducted by telephone and, due to COVID-19 restrictions, will be administered by the PI by telephone, who has been certified in the administration and scoring of the MoCA. Potential participants will be considered eligible to participate in the study if: their GAI score is ≤ 10; and their T-MoCA score is ≤ 19 [37, 38]. The T-MoCA is conducted by telephone and is a validated version of the MoCA that assesses phonemic fluency; word recall and orientation to day, date, and location; and does not use items that require paper and pencil or animal identification [38, 39]. The T-MoCA is demonstrated to be accurate in detecting mild cognitive impairment (95% confidence interval) with excellent test-retest reliability and internal consistency [38, 39].

### Interventions

#### Comparators

The comparator will be no treatment (treatment as usual) for the controlled wait list group. All participants will receive the intervention. All participants will receive T1, T2, and T3 assessments within 7–10 days of each timepoint.

#### Intervention description

EFMT is a group intervention traditionally consisting of eight weekly in-person group meetings of 2.5 h, with group sizes of between 10 and 12 participants. Due to COVID-19 restrictions, EFMT will be delivered by Zoom to groups of six participants and weekly meetings will be shortened to 1.5 h to reduce Zoom fatigue, a phenomenon noted in popular media [40, 41]. For modification to video-delivery of the intervention, the number of participants has been decreased from 10 to 12, to six to provide adequate time for all components of the intervention to be delivered. Each group meeting consists of a check-in period of 5 min, the reading of meditation instructions by the PI, 20 min of silent unguided meditation, 5–10 min of journaling about what arose for participants during the meditation, and a 5-min break. After journaling, each participant takes a turn and describes their meditation experience, with the PI offering empathic exploration. In emotion-focused mindfulness therapy, as in emotion-focused therapy, empathic exploration is a core competency that involves reflection, validation, responding to participants, and heightening their emotional experience [42, 43]. This is the process that is the heart of change and encourages participants to become more familiar with, and develop a tolerance to, distressing emotions [33].

EFMT poses little risk for participants. Meditation can sometimes cause uncomfortable feelings to arise when participants connect with emotions. Participants may decide to withdraw from the intervention if overwhelming emotions emerge. Participants may decline to answer questions during the reporting part of the meditation group. Should a participant become upset during any part of the group, the PI can: ask that participant to remain after the group to talk; ask that group participants take a short break while the PI speaks with the upset participant; lead the upset participant through a relaxation and grounding exercise; ask the participant if the PI may contact someone of their choice for support; and help the upset participant to access counseling. Participants may withdraw from the study at any time; however, study participants will be retained in the trial wherever possible to enable follow-up data collection and prevent missing data.

### Outcome collection and measures

*Aim 1*. Determine if participants can be recruited, enrolled and retained

Recruitment, enrollment and retention data will be used to evaluate feasibility. Data will be gathered and measured on the number of potential participants referred and screened for eligibility, participants meeting eligibility requirements, participants enrolled, and participants completing the intervention. Recruitment and enrollment will be considered feasible if 70% of the total number of potential participants meeting eligibility requirements consent to enroll [44], and if 48 participants can be enrolled within a 3-month period (16 participants recruited per month). Retention will be considered feasible if 65% of participants complete data collection at both timepoints [45].Variables such as changes to medications, beginning, or stopping mental health counseling and significant life events will be tracked by the RA at T3 data collection timepoint. Extraneous variables will be important to gather to identify other potential reasons for changes to secondary outcomes aside from EFMT.

*Aim 2*. To determine if participants can adhere to all components of the intervention

Adherence will be used to measure acceptability. The intervention will be considered acceptable if 70% of participants attend of a minimum of six meetings [44]. As well, acceptability for measures of adherence to meditation, journaling, and reporting will be gathered through PI observation and documentation. Adherence to these components will be considered acceptable if the majority (75% or more) of participants appear to be meditating, journaling and actively report their meditations. Participants will not be required to practice between meetings; however, data will be gathered on the frequency and duration of home practice meditation and journaling to explore whether there is a relationship to outcomes. Participants will complete weekly reporting forms to be emailed to the PI. The PI will keep an observational diary noting what works well and perceived areas of challenge.

*Aim 3*. To determine the secondary objective of whether participants who receive the intervention potentially have decreased levels of anxiety compared to those participants who do not receive the intervention, the GAI will be used [35]. Outcome measures will be collected using the GAI at T1, T2 and T3.

**Fig. 1:**
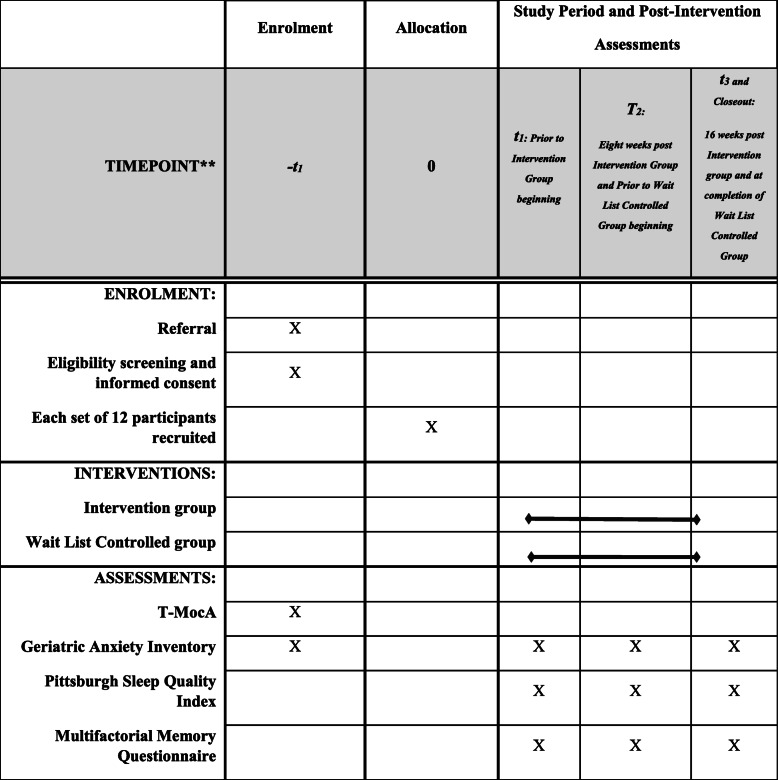
Standard Protocol Items: Recommendations for Interventional Trials (SPIRIT) Schedule of enrolment, intervention and assessments. T1 data collection: RA1 will collect demographic data and T1 outcome measures will be administered individually by telephone. Participants will be informed that they will be notified about the start date of their group. T2 and T3 post intervention data collection: At the end of the first intervention, all participants will be contacted by telephone to conduct individual T2 assessments. For T3 data collection, all participants will be contacted by telephone by RA1 to conduct individual assessments

To determine the secondary objectives, the extent to which participants who receive the intervention potentially experience changes to sleep quality and subjective memory functioning compared to participants who do not receive the intervention, the Pittsburgh Sleep Quality Index (PQSI) and the Multifactorial Memory Questionnaire (MMQ) will be used [46, 47] (Fig. 1).

### Sample size justification

A sample size of 48 participants will allow for intervention group sizes of six participants and wait listed control group sizes of six participants. There will be four blocks of participants, with 12 participants in each block, and groups of six participants. This will result in 24 participants per arm. Small sample sizes of 10–20 participants or more are appropriate for pilot testing of adherence to interventions and this study will exceed those numbers, thereby allowing for attrition [48, 49]. There are also pragmatic considerations to selecting the sample size for this study [50], which include planning to complete the study within a year with limited funding. The study will not be powered to detect significant changes on the secondary outcomes. As COVID-19 protocols prohibit groups meeting in person, this study has moved to Zoom, an online privacy protected platform [51]. Other COVID-19 adjustments pivots required will be shorter meetings of 1.5 h to avoid Zoom fatigue and smaller group sizes [40]. This will be sufficient to meet the study’s primary objectives of recruitment, retention, and completion rates to determine whether a large study is warranted.

### Recruitment

Adults 55 years of age and older will be recruited through two primary care clinics in Eastern Ontario, Ontario-based community organizations that support older adults, and community referrals through traditional snowball effect [52]. The PI will screen potential participants for eligibility. The following participant characteristic data will be collected by the PI at the time of enrolment into the study: age, gender, working status, educational status, marital status, living situation, and how many times, if at all, the participant presently meditates per week. Referrals to the PI will be made by health care providers, staff at community-based organizations supporting older adults, and through community spread. Referees and self-referring community-based potential participants will be informed of the study through email, advertisements on a local radio station’s website, and social media. All costs associated with recruitment will be self-funded by the PI, a PhD candidate. Duration of recruitment will be monitored by the PI and her PhD advisors. No financial incentives will be offered to participants. Potential benefits to participants will be receipt of instruction in a mindfulness-based meditation therapy group. The possible benefit to society at will be to determine the feasibility of a scalable treatment for older adults with anxiety.

### Randomization

Random allocation will be conducted by the second author, immediately after each set of 12 participants has been enrolled into the study by the PI*.* Groups will begin on a rolling basis each time a block of 12 participants has been enrolled. To ensure a 1:1 random allocation between the intervention and control groups, the participants will be randomly assigned using the random allocation function in Excel.

### Blinding

RA will be blinded to participant allocation to ensure unbiased assessment administration and data collection throughout the study. Participants will be masked from the group to which they have been allocated. The PI will remain concealed to the outcome measurements data until all participants have completed the intervention and statistical analysis has begun. Unblinding and unmasking may unintentionally occur. It is possible that the RA may be unblinded to the allocation of participants who may unintentionally reveal their assignment to group during the collection of T2 outcome measures. This is a pragmatic reality of conducting a small scale self-funded PhD-based study.

### Data collection and management

#### Collection of information at T1

T1 data collection will be conducted by the RA, a master’s level researcher trained by the PI in the administration of scoring of the three assessments to be administered. The RA will administer the GAI. The GAI has been validated for use in community dwelling adults over the age of 60, with excellent test-retest reliability of *r* = .91 to .95 [35, 53–55]. This anxiety scale was selected because it is commonly used in a range of clinical settings [56]. The RA will administer the PSQI and the MMQ [46, 57]. The PQSI is a sleep quality and sleep disturbance assessment validated for use with community dwelling older adults [46]. This measure was selected because sleep complaints are frequently associated with anxiety [58]. The PQSI is a simple 19 item subjective measure of sleep quality and patterns of sleep using a 0 to 3 Likert scale [59]. The PQSI has been demonstrated to have excellent test-retest reliability (*r* = .85–.87) and internal consistency of Cronbach’s *a* of .80–.83 [46, 60, 61]. The MMQ is a 21-item brief self-report assessment of subjective memory ability validated for use with community dwelling older adults [57, 62]. This assessment measures satisfaction, ability and strategy use in memory. It was chosen because older adults with anxiety frequently report problems with memory [4, 5, 15–17]. The MMQ has high test-retest reliability over time (.86–.95) [62, 63]. Cronbach’s *a* internal consistency is good at .87 to .93 [57, 62, 64].

#### Collection of information at T2

All participants will be contacted by telephone by the RA to re-administer the GAI, PSQI, and MMSQ within 1 week of completion of the intervention.

#### Collection of information at T3

All participants will be contacted by telephone by the RA to re-administer the GAI, PSQI, and MMSQ and to collect data on extraneous life events, within 1 week of completion of the intervention for the wait list control group. The RA will attempt to collect data from all participants whether or not all sessions are attended.

### Feasibility and acceptability outcomes

Feasibility and acceptability outcomes will be determined by the following: recruitment and enrollment will be considered feasible if 70% of the total number of potential participants meeting eligibility requirements consent to enroll [44], and if 48 participants can be enrolled within a 3-month period (16 participants recruited per month). As a dropout rate of 35% is consistent with MBIs based in self-management and with a technology based delivery, a retention rate of 65% will be used to determine feasibility [65, 66]. Participants’ adherence to all components of the intervention will be defined by attendance of 6/8 sessions and participation in meditation, journaling, and reporting during the sessions, as observed and documented by the PI. In the tradition of MBI research, the intervention will be considered complete if participants are able to attend a minimum of 6/8 meetings [45]. It will be assumed that participants will be honest about meditation within the group, although direct evidence can only be inferred through observation [67]. Participants are not required to practice at home; however, they will be asked to self-report on the frequency and duration of home practice meditation and journaling to explore whether there is a relationship to outcomes.

### Treatment fidelity

The PI received training and certification in a joint program with the University of Toronto, Faculty of Medicine, and Mt. Sinai Hospital, Toronto, Canada.

### Participant retention

The PI will encourage participant retention by sending reminder emails of group start dates to wait listed participants at 4 weeks, 2 weeks, and 1 week prior to the beginning of the group.

### Statistical methods

Data entry will be conducted by the RA. Data analysis will be conducted by the PI at the end of the study using SPSS V27 [68]. All data will be examined by running frequencies on each variable to determine if values are missing or out of range. Possible errors will be compared to raw data and corrected as necessary to ensure accuracy [67]. As is recommended for pilot feasibility studies, the following descriptive statistics will be used: for continuous variables, minimum, maximum, mean, median, mode standard deviation, skewness, and kurtosis; if continuous variables are skewed, non-parametric descriptive statistics will be reported; for categorical variables, frequencies will be used [69, 70]. Graphical displays such as histograms or boxplots will be presented to demonstrate and better understand the central tendency and dispersion of the data [67]. Recruitment, enrollment, and retention rates will be calculated. Rates of attendance and adherence to each component of the intervention will also be calculated to determine acceptability.

To examine whether there have been potential changes in the secondary outcomes, descriptive statistics will be used to analyze and present demographic and baseline data. Categorical and ordinal data will be compared using chi-square. Continuous data will be compared using a *t*-test. The mean change in the intervention group and wait list control group outcomes will be compared post intervention (T3) using an independent samples *t*-test, and Cohen’s *d* will be calculated for effect size [67]. Data from each of the groups will be combined to form a larger sample size to evaluate the effect of the intervention. Analysis of pooled data will be conducted using a paired sample *t*-test and Cohen’s *d* to examine the intervention effect over baseline measures. Change over time for the intervention group from T1 to T2 to T3 will be calculated by using repeated measures ANOVA to determine if change has occurred and/or is maintained in the intervention group.

### Data management

All data forms will be uploaded to the Microsoft Teams environment, password protected, and backed up in the Queen’s University cloud storage. The RA will enter all data in SPSS [68]. Data will be kept for seven to 10 years after. Data will be held in a secure environment on Canadian servers in the Queen’s University research data repository for faculty, students, and staff [65].

### Confidentiality

All information collected about potential participants will be destroyed should eligibility not be met for the study. All data collected from participants will be identified by a study identifier only to maintain participant confidentiality. No identifying information will be included when results of the study are shared. Participants will be asked to maintain confidentiality of other participants such as identifying information and meditation reflections. No record of the Zoom meetings will exist.

### Auditing trial conduct

This trial will be audited by the second author for completeness and accuracy of data collection and retention of participants across arms of this study.

### Ethics and dissemination

This trial has been approved by the Queen’s University Health Sciences & Affiliated Teaching Hospitals Research Ethics Board (approval number REH-768-20). This clinical trial has been registered at ClinicalTrials.gov for accessibility by other researchers. This research study received the written support of the Prince Edward Family Health Team and the Queen’s Family Health Team. The PI will inform the PhD committee of any changes to the protocol such as recruitment procedures, intervention, or data collection and analyses. As appropriate, amendments will be requested of Queen’s University HSREB and approvals received prior to any changes to this protocol. Protocol amendments will be communicated via ClinicalTrials.gov (ID NCT04415528) updates. Results will be disseminated through peer reviewed journals, international conferences, and primary care grand rounds.

### Consent

Prior to screening for eligibility, each potential participant will receive an emailed copy of the Letter of Information/Informed Consent and will provide informed consent to the PI at the beginning of the screening call (Additional file 1). Data gathered from screened potential participants not eligible to participate will be destroyed. The reason for ineligibility will be noted. All data will be anonymized. In the event of withdrawal from the study, the participant will have the choice of requesting that collected data be destroyed or to give consent to include data for analysis.

The purpose of this protocol is to ensure transparency and reproducibility of the results of this study. Therefore, this protocol will be available publicly through journals and trial registries [66, 71]. The deidentified data will be archived to Queen’s University Scholars Portal Dataverse, a repository for research data that is stored on Canadian servers in a secure environment, within 3 years of collection and will be available for sharing [72].

## Discussion

A potential limitation to this trial is that it is not powered to examine effectiveness and findings will not be generalizable to other populations. However, findings will inform planning of a definitive RCT including anticipated time required for recruitment, strategies to promote adherence, and sample size calculations. This study will not examine hypothesis testing based on the CONSORT extension. Due to COVID-19, this group has been modified for delivery in an online format to ensure the participants’ safety. This trial will provide unique information to international researchers and clinicians regarding a novel treatment for late-life anxiety and will become the first RCT to determine the feasibility of EFMT for older adults with late-life anxiety.

## Conclusion

Significant rates of anxiety among older adults can be expected to increase as the world population ages and within the COVID-19 and post COVID-19 environment [24, 73, 74]. Anxiety is a leading cause of disability in older adults [75]. EFMT is a low-cost non-pharmacological treatment that could be offered in primary care and community settings. EFMT has the potential to provide older adults with the knowledge, skills, and practice to reduce anxiety.

## Supplementary Information

**Additional file 1.**

## Data Availability

Data will be kept for 7 to 10 years after it is analyzed and published. Data will be held in a secure environment on Canadian servers in the Queen’s University Dataverse, a research data repository for faculty, students and staff [65].
