# Supplementary material for: Video-delivered emotion-focused mindfulness therapy for late- life anxiety: study protocol for a feasibility randomized controlled trial
Source: Pilot Feasibility Stud. 2021 Sep 3;7:169. doi: 10.1186/s40814-021-00905-0 (PMC8413357; doi:10.1186/s40814-021-00905-0)
Supplement: Supplementary file 1 — Additional file 1. [file 40814_2021_905_MOESM1_ESM.docx]

**Appendix A: Letter of Information and Informed Consent Given to Participants**


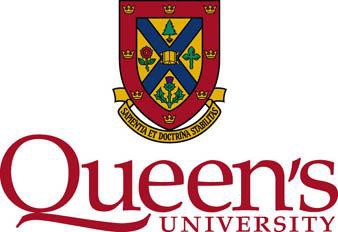


**Participant Information Sheet and Consent Form**

**Emotion-Focused Mindfulness Therapy: A feasibility study of an intervention for late life anxiety**

**Researchers:**

Ms. Stacey Hatch, PhD candidate, Aging & Health,

phone: 613-476-0400 x 217

email: [shatch@pefht.com](mailto:shatch@pefht.com)

supervised by Dr. Marcia Finlayson and Dr. Dorothy Kessler, both of the School of Rehabilitation Therapy, Queen’s University, Kingston, Ontario.

**Introduction:**

You are being invited to take part in this research project because you are 55 years of age or older and are experiencing symptoms of anxiety.

Please read this Participant Information Sheet and Consent Form carefully and ask as many questions as you like before deciding whether to take part in this research study. We will go over this form together today. You can discuss this decision with your family, friends and your health care team.

**Background, purpose and design of the study**

About 29% of Eastern Ontario adults aged 65 and older have symptoms of anxiety. Emotion-Focused Mindfulness Therapy (EFMT) is an eight-week meditation program that may reduce symptoms of anxiety in adults. While these eight-week groups typically meet in person for about 2.5 hours, due to COVID-19 restrictions, this group may be modified to meet in an on-line platform for about 1.5 hours, to ensure your safety. The purpose of this study is to test whether EFMT is acceptable to adults who are 65 and older and have symptoms of anxiety.

Each group meeting involves a silent meditation of about 20 minutes, followed by about 5 to 10 minutes of journaling about what came up for you in the meditation. In this type of meditation, you are free to consider the future, or remember and reflect on past experiences while meditating. After journaling each participant will be asked questions about their meditation. Being asked to reflect on our meditation deepens the experience of what arose for us in a mindfulness way.

**Study Procedures**

You will have a telephone conversation with Stacey Hatch. This conversation will take about 30 minutes. The purpose of this call is to screen you for eligibility in the study. She will ask you to complete an assessment called the General Anxiety Inventory (GAI). This assessment asks questions about how anxiety impacts your daily life. This assessment takes about 10 minutes to complete. Next, she will ask you to complete a Telephone Montreal Cognitive Assessment (T-MoCA) to determine your level of memory functioning. This assessment takes about 10 minutes.

If you are not eligible for the study, the information collected during the screening phone call will be immediately destroyed in a secure manner. There will be no record of any information that you provided.

If you are eligible for the study and decide to participate, the following describes next steps. A research assistant (RA) will call you within one month. The RA will ask you to complete the GAI again, plus two more brief assessments: the Pittsburgh Sleep Quality Index (takes about 5 minutes to complete) and the Multifactorial Memory Questionnaire (takes about 15 minutes to complete). You will be informed about whether you will be in the group that begins immediately, or whether you will be placed on a wait list for the group that will begin in eight weeks. If you are wait listed, you will be contacted by the RA in eight weeks, before you begin the group, to retake the assessments. You will also be contacted by the RA eight weeks after the end of your group to retake the assessments. These calls will take about 20 minutes each.

During the study, you will also be asked to complete a weekly on-line reporting form, which will take less than five minutes to complete.

**Potential risks and discomforts**

The risks to taking part in this study are small. Meditation can sometimes cause uncomfortable feelings to arise when you connect with emotions. There is a risk that some questions asked during the reporting part of could upset you. You can refuse to answer questions during the reporting part of the meditation group. If you do become upset during any part of the group, I will ask you to stay after the group to talk, or the group will take a short break while we chat. I may lead you through a relaxation and grounding exercise, ask your permission to notify someone that you choose or help you access counselling. If you become upset after the group or between group meetings, I can help you access counselling if you think this would be helpful to you.

**Possible benefits**

There are no known benefits to you for taking part in this study. The potential benefit to you is that you will receive instruction in a new type of mindfulness meditation in a group setting. This setting may be in person or it may be online, depending on COVID-19 restrictions. You may find that your symptoms of anxiety reduce. There is also a potential benefit to society. Eastern Ontario has high rates of anxiety in adults who are 55 years of age and older, and this may be a potential treatment to help ease some of these symptoms of anxiety.

**Withdrawal from the study**

You have the right to leave the group at any point. You have the right to refuse to answer any questions or share personal information during the reporting component of the groups. You may refuse to answer any questions you do not want to answer and remain in the study. Your participation in this research is entirely voluntary. Your decision to participate or withdraw will not affect any relationship, current or future, you have with Queen’s University or the primary care provider who referred you to this study.

You may cancel this consent at any time. If you withdraw your consent, you may choose whether or not you want to have the information collected from you so far included in the study.

**Study costs**

You will not be paid to participate in this study.

**Confidentiality**

We will keep the results of your assessments confidential. The conversations we have during the groups will not be recorded. Your privacy and identity will be protected by assigning a code rather than your name to your answers. The investigators (Mrs. Stacey Hatch, Dr. Finlayson, Dr. Kessler and a Research Assistant) will have access to your information. The Queen’s University and Affiliated Teaching Hospitals Research Ethics Board (HSREB) may require access to study-related records to monitor the ethical conduct of the research.

When we share the results of this study, no information will be included that would reveal your identity. We will identify you by a participant number, not your name, in order to maintain the confidentiality of your data. Any information that you provide through the study will remain confidential.

All the study information collected from you will be stored in a safe and locked location. Data will be kept for seven to ten years after it is analyzed and published. Your name will not appear on any documents related to your participation in this study.

As a participant, your confidentiality will be protected by the research team.

**Study results**

The results of this study will be shared with the public to advance knowledge about EFMT. The results will be published in an academic journal. Results of this study will also be shared with other researchers through conferences and webinars. Healthcare professionals will also be informed about the results of this study through meetings, webinars and publication of the study results. If you decide to participate, you will receive an emailed copy of the published academic articles about this study.

The investigators (Stacey Hatch, Dr. Finlayson and Dr. Kessler) will have access to this information. We will identify you by a participant number, not your name, in order to maintain the confidentiality of your data. Any information that you provide through the study will remain confidential. All the study information collected from you will be stored in a safe and locked location. Data will be kept for seven to ten years after it is analyzed and published. Your name will not appear on any documents related to your participation in this study.

**If you have concerns**

If you have any questions about this study, you may contact either Dr. Marcia Finlayson at 613-533-2576, or [marcia.finlayson@queensu.ca](mailto:marcia.finlayson@queensu.ca) or Dr. Dorothy Kessler at 613 533 6551, or dk75_queensu.ca. have any questions about the ethics of this study, you may contact the Queen’s University Health Sciences and Affiliated Teaching Hospitals Research Ethics Board (HSREB) at 1-844-535-2988 (Toll free in North America).

*This study has been reviewed for ethical compliance by the Queen’s University Health Sciences and Affiliated Teaching Hospitals Research Ethics Board.*


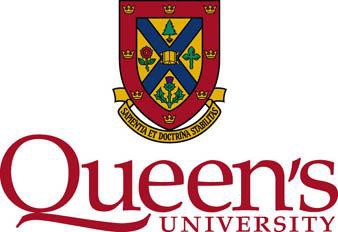


**Consent Form**

**Project title**: **Emotion-Focused Mindfulness Therapy: A feasibility study for late life anxiety**

1. I have read the Letter of Information and have had any questions answered to my satisfaction. I understand that I am being invited to participate in a research study called “Emotion-Focused Mindfulness Therapy: A feasibility study for late life anxiety”.
2. I understand that if I agree to participate, I will be asked to participate in three telephone interviews, and eight weekly group meetings of Emotion-Focused Mindfulness Therapy, either in person or by video conference platform.
3. I understand that my participation in this study is voluntary and I that I may withdraw at any point.
4. I understand that every effort will be made to maintain the confidentiality of my data now and in the future. Only the investigators (Ms. Stacey Hatch, Dr. Marcia Finlayson and Dr. Dorothy Kessler) and the research assistant will have access to this information. The team will publish the findings in professional journals and present them at scientific conferences and community events. Findings will be combined across all participants and will never breach individual confidentiality. Should I be interested, I am entitled to a copy of the findings.
5. I am aware that Ms. Stacey Hatch, as a member of the *College of Registered Psychotherapists of Ontario*, has a legal duty to warn or report (an obligation to disclose certain confidential information without consent) incidents of suspected child abuse or neglect, or where information exists suggesting an immediate risk of self-harm or severe threat to an identifiable person.
6. As a participant in this research I understand that I have not waived any legal rights and have been provided a copy of this letter.

I am aware that if I have any questions, concerns, or complaints, I may contact Dr. Marcia Finlayson at 613-533-2576 or [marcia.finlayson@queensu.ca](mailto:marcia.finlayson@queensu.ca) or Dr. Dorothy Kessler at 613 533 6551, or dk75_queensu.ca. have any questions about the ethics of this study, you may contact the Queen’s University Health Sciences and Affiliated Teaching Hospitals Research Ethics Board (HSREB) at 1-844-535-2988 (Toll free in North America).

I verify that: I have read the *Letter of Information and the Consent Form* and all of my questions have been answered. A copy of this letter has been provided for my records. I freely consent to participate in this research.
